# Chromosomal Instability and Periodontal Disease in Idiopathic Infertility: Evidence of a Possible Association

**DOI:** 10.3390/biology14091256

**Published:** 2025-09-12

**Authors:** Cristina-Crenguţa Albu, Ştefan-Dimitrie Albu, Claudia Florina Bogdan-Andreescu, Cristian-Viorel Poalelungi, Constantin Marian Damian, Alexandru Burcea, Andreea-Mariana Bănățeanu, Emin Cadar, Dan Alexandru Slăvescu, Anca Daniela Brăila

**Affiliations:** 1 Department of Genetics, Faculty of Dentistry, “Carol Davila” University of Medicine and Pharmacy, 020021 Bucharest, Romania; 2 Department of Periodontology, Faculty of Dentistry, “Carol Davila” University of Medicine and Pharmacy, 020021 Bucharest, Romania; 3 Department of Speciality Disciplines, Faculty of Dentistry, “Titu Maiorescu” University, 031593 Bucharest, Romania; 4 Department of Obstetrics and Gynecology, Faculty of Medicine, “Carol Davila” University of Medicine and Pharmacy, 020021 Bucharest, Romania; 5 Department of Obstetrics and Gynecology, University of Medicine and Pharmacy of Craiova, 200349 Craiova, Romania; 6 Faculty of Pharmacy, “Ovidius” University, 900470 Constanța, Romania; 7 Department of Dentistry, Faculty of Medicine and Pharmacy, University of Oradea, 410073 Oradea, Romania

**Keywords:** chromosomal instability, infertility, periodontitis

## Abstract

Background: Chromosomal instability (CIN) may underlie a subset of idiopathic infertility, and chronic periodontitis could contribute to genomic fragility. We tested whether periodontal status is associated with cytogenetic instability in adults with idiopathic infertility. Methods: This was a cross-sectional study of 60 adults aged 20–40 years, comprising idiopathic infertility (*n* = 30) and fertile controls (*n* = 30), each with 18 women and 12 men. Significant exclusions included systemic inflammatory disease, pregnancy/lactation, recent antibiotics/NSAIDs, and periodontal therapy within 6 months. Periodontal examination recorded probing depth (PD), clinical attachment loss (CAL), and bleeding on probing (BOP). Cytogenetic testing used mitomycin C-induced chromosomal breakage to derive the Breakage Index (BI); CIN was defined as BI ≥ 4.0. Analyses compared infertile with CIN (*n* = 19), infertile without CIN (*n* = 11), and controls (*n* = 30). Results: Infertile participants with CIN had a higher periodontitis burden compared to infertile participants without CIN and to controls (moderate–severe: 89.5% vs. 54.5% vs. 26.7%); mean BI also differed (5.2 ± 0.9 vs. 1.3 ± 0.5 vs. 0.4 ± 0.2). Periodontal measures followed the same gradient, with greater CAL and PD in CIN-positive infertility. Conclusions: In idiopathic infertility, CIN was cross-sectionally associated with more severe periodontitis, and the BI correlated with CAL, PD, and BOP. Causality cannot be inferred and residual confounding cannot be excluded. Periodontal screening is a feasible adjunct that may help identify a modifiable inflammatory burden; prospective and interventional studies are warranted.

## Simple Summary

We studied 60 adults aged 20–40 years: 30 with idiopathic infertility and 30 fertile controls, each cohort balanced by sex (18 women/12 men). Every participant underwent a comprehensive periodontal examination and a blood cytogenetic test that summarizes chromosomal fragility as the Breakage Index (BI). Among infertile participants, those with chromosomal instability had more moderate-to-severe periodontitis compared to infertile participants without instability and to fertile controls. Standard periodontal measures—clinical attachment loss, probing depth, and bleeding on probing—were consistently aligned with BI gradients. Because this was a cross-sectional study, it does not establish cause and effect; however, these findings suggest that adding periodontal screening to infertility care could help identify a modifiable inflammatory burden relevant to overall health.

## 1. Introduction

Infertility is clinically defined as the inability to conceive after 12 months of regular, unprotected sexual intercourse [1]. Its prevalence ranges from 10% to 15% globally, with higher rates in certain industrialized countries or environmentally stressed populations [2]. The causes of infertility are diverse and related to male and/or female factors, and include anatomical, hormonal, infectious, and genetic anomalies [3,4]. However, in approximately 15% of cases, no clear cause is identified even after comprehensive investigations, leading to the diagnosis of idiopathic infertility [5].

Among the less frequently explored mechanisms in idiopathic infertility is chromosomal instability (CIN), a condition characterized by an increased rate of chromosomal aberrations, including breaks, gaps, translocations, and aneuploidies [6]. CIN may affect both somatic and germ cells, with profound consequences on gametogenesis, fertilization, and early embryonic development [7]. Traditional karyotyping can detect major abnormalities, but subtle chromosomal fragility often requires specialized assays, like mitomycin C-induced chromosomal breakage tests, to expose latent genomic instability [8].

At the same time, periodontitis is a chronic, multifactorial inflammatory disease of the supporting structures of the teeth [9]. It is initiated by bacterial biofilms and sustained by an exaggerated host immune response, resulting in the progressive destruction of the periodontal ligament and alveolar bone [10]. The systemic impact of periodontitis has been extensively documented, including association with cardiovascular pathology, insulin resistance, and adverse obstetric outcomes [11]. The molecular mechanisms by which periodontitis exerts systemic effects involve the dissemination of inflammatory cytokines (e.g., IL-6, TNF-α), bacterial endotoxins, and reactive oxygen species (ROS), all of which have the potential to damage DNA and impair genomic integrity [12].

Recent clinical and experimental studies have proposed a possible link between periodontal disease and infertility. For instance, a case–control study by Nwhator et al. demonstrated significantly increased odds of conception within one year in women with good oral hygiene compared to those with periodontal inflammation [13]. Likewise, Hart et al. reported that periodontal disease was associated with an average two-month delay in conception, with stronger effects in non-Caucasian populations [14]. Despite these findings, awareness among reproductive health professionals remains limited. A survey by Nwhator et al. revealed that very few fertility screenings routinely inquire about oral health or refer patients for periodontal evaluation [15].

Preclinical data support this association: in murine models, Arce et al. demonstrated that infection with periodontal pathogens, such as *Campylobacter rectus* and *Porphyromonas* gingivalis, led to decreased fetal weight, increased placental necrosis, and a higher rate of fetal resorption [16]; Similarly, Fogacci et al. showed that ligature-induced periodontitis in rats reduced fecundity and elevated inflammatory cytokines at both local and systemic levels [17]. However, human studies report inconsistent associations between periodontitis and birth-weight-related outcomes, as well as infertility [18,19,20]. Notably, the present study did not evaluate pregnancy or neonatal endpoints (e.g., birth weight).

While these findings highlight the role of inflammation in reproductive dysfunction, recent cytogenetic research has suggested that genomic instability may serve as a key intermediary mechanism. Several studies have documented increased frequencies of micronuclei—established markers of chromosomal damage and mutagenic events—in exfoliated oral epithelial cells of patients with chronic periodontitis [21,22,23,24,25]. Benedek, Tadin et al. reported significantly elevated micronucleus counts in gingival cells from individuals with advanced periodontal inflammation [21,22], while Alkan, Muradyan et al. associated these cytogenetic abnormalities with smoking and periodontal status [23,24]. The HUMN project has validated the buccal micronucleus assay as a sensitive and non-invasive method to monitor environmental and pathological genotoxic exposures [25]. These findings suggest that chronic oral inflammation may not only affect local tissues but may also contribute to systemic genomic instability, potentially compromising reproductive chances.

Given the biologically reasonable mechanism and shared inflammatory pathways, it could be hypothesized that chronic periodontitis may represent a risk factor for CIN [26,27,28]. Collectively, these observations support the hypothesis that this systemic connection may contribute to the pathophysiology of idiopathic infertility by promoting oxidative DNA damage and genomic fragility [29].

This study aims to investigate the potential relationship between CIN and chronic periodontitis by combining clinical periodontal assessments with cytogenetic and molecular analyses in a selected cohort of infertile patients and healthy controls.

## 2. Materials and Methods

### 2.1. Ethical Considerations

This observational, cross-sectional study was conducted over 12 months at a multidisciplinary academic medical center. Ethical approval was obtained from the Institutional Review Board (IRB approval no. 48/2021), and all participants provided informed written consent in accordance with the Declaration of Helsinki.

### 2.2. Participants and Eligibility Criteria

Sixty adults, aged 20–40 years, were recruited and divided into two equal groups. Group A (infertility group) included 18 women and 12 men diagnosed with idiopathic infertility, while Group B (control group) included 18 women and 12 men who had conceived naturally within the past three years.

The study group (Group A) comprised 30 patients diagnosed with idiopathic infertility, defined as the absence of conception despite regular unprotected intercourse for at least one year, with no identifiable anatomical, hormonal, infectious, or endocrinological causes. The control group (Group B) included 30 age- and sex-matched fertile individuals who had conceived naturally within the past three years and had no history of infertility or systemic disorders.

Inclusion criteria for both groups were: age 20–40 years; absence of systemic chronic inflammatory conditions (e.g., diabetes, autoimmune diseases); non-smokers or light smokers (<10 cigarettes/day); no systemic antibiotic or anti-inflammatory (NSAID) therapy within the previous 30 days; and no periodontal treatment within the previous six months. At enrollment, a brief eligibility interview was conducted to document diet, oral hygiene, physical activity, supplementation, and a complete medication history (including all prescription and over-the-counter drugs) for the preceding three months. These data were used solely to confirm eligibility and to describe the cohort.

Exclusion criteria included known chromosomal disorders, immunosuppressive therapy, malignancy, pregnancy, or lactation. In addition, participants with current or recent use (past 3 months) of medications known to affect periodontal tissues or bone metabolism or systemic immunity were excluded, including systemic corticosteroids, immunosuppressants (e.g., calcineurin inhibitors), antineoplastic agents, antiresorptives (bisphosphonates or denosumab), phenytoin, calcium-channel blockers associated with gingival overgrowth (e.g., nifedipine), cyclosporine, and oral retinoids (e.g., isotretinoin). Participants who had taken short courses of antibiotics or NSAIDs in the previous 30 days were rescheduled and reassessed outside these windows.

Both women and men were enrolled to enhance external validity and to allow exploratory, sex-stratified assessment of periodontal and cytogenetic outcomes. The complete inclusion and exclusion criteria are summarized in Table 1.

### 2.3. Cytogenetic and Molecular Assessment

From each participant, 5 mL of peripheral venous blood was collected in sterile heparinized tubes and processed within four hours of collection. Lymphocyte cultures were initiated in RPMI-1640 medium enriched with 20% fetal bovine serum, L-glutamine, a standard antibiotic mixture, and 2% phytohemagglutinin-M to stimulate mitosis. Two parallel cultures were established per subject: a baseline culture and a mitomycin C (MMC)-stimulated culture, with MMC added at a final concentration of 0.01 μg/mL for the last 24 h [30].

After a 72-h incubation period at 37 °C with 5% CO_2_, metaphase chromosome spreads were prepared by hypotonic treatment (0.075 M KCl), fixation with methanol: acetic acid (3:1), and staining following the Giemsa protocol. At least 20 high-quality metaphases were analyzed per subject using a digital metaphase analysis platform. Structural chromosomal aberrations (e.g., chromatid breaks, chromosomal gaps, triradials, dicentrics) were identified and recorded [30].

The degree of CIN was quantified using the Breakage Index (BI), calculated as the total number of observed chromosomal aberrations divided by the number of metaphases scored. A BI ≥ 4.0 was considered indicative of significant genomic instability [31]. In selected cases, array-based comparative genomic hybridization (aCGH) and polymerase chain reaction (PCR) assays targeting Y-chromosome microdeletions (AZFa, AZFb, AZFc) were used to rule out submicroscopic chromosomal abnormalities [32].

### 2.4. Periodontal Examination

Comprehensive periodontal evaluation was conducted by a board-certified periodontist who was blinded to the cytogenetic results. Full-mouth clinical examinations were performed, excluding third molars, and included six-point measurements per tooth using a calibrated UNC-15 periodontal probe (Hu-Friedy, Chicago, IL, USA). The clinical parameters recorded were: Probing Depth (PD), Clinical Attachment Loss (CAL), Bleeding on Probing (BOP), and Plaque Index (PI). PD was recorded as the distance from the gingival margin to the base of the sulcus/pocket; CAL was derived relative to the cemento-enamel junction (accounting for gingival recession); BOP was recorded as the percentage of sites bleeding within 15 s after gentle probing; PI was assessed using the Silness–Löe index (0–3 per surface, averaged per patient).

The diagnosis and staging of periodontitis were made according to the 2018 classification system jointly proposed by the American Academy of Periodontology (AAP) and the European Federation of Periodontology (EFP) [33]. Disease staging was based on the extent and severity of CAL, radiographic bone loss, and complexity factors, including tooth mobility and occlusal dysfunction [33].

To ensure inter-rater reliability, calibration exercises were conducted before the study, and intra-examiner agreement was confirmed using the intraclass correlation coefficient (ICC), which exceeded 0.85 for all clinical parameters. Radiographs were obtained as needed for bone level assessment. All measurements were entered into a standardized data collection platform for further statistical analysis.

### 2.5. Statistical Analysis

Statistical analyses were performed using IBM SPSS Statistics for Windows, version 28.0 (IBM Corp., Armonk, NY, USA) and GraphPad Prism, version 10.1.1 (GraphPad Software, San Diego, CA, USA). Continuous variables were summarized as mean ± SD or median (IQR), as appropriate; categorical variables as counts and percentages. All tests were two-sided with α = 0.05, and results were interpreted alongside 95% confidence intervals (CIs).

Group comparisons. Two-group contrasts used Welch’s *t*-test (allowing unequal variances) or Mann–Whitney U when normality was not met. Three-group contrasts used Welch ANOVA with Games–Howell post hoc tests or Kruskal–Wallis with Dunn’s post hoc tests, as appropriate. Proportions were compared using χ^2^ or Fisher’s exact tests.

Assumptions and effect sizes. Normality was assessed with Shapiro–Wilk and Q–Q plots; variance homogeneity with Levene’s test. Continuous outcomes are reported as mean differences with 95% CIs (Welch/Satterthwaite) and Hedges’ g (small-sample corrected). For proportions, we report risk difference (RD) with Newcombe 95% CI (Wilson score method).

Associations and figures. Relationships between breakage index (BI) and periodontal parameters (CAL, PD, BOP) were evaluated visually and summarized through group-wise contrasts (Table 2). Graphical outputs (bar charts, scatter plots, heatmaps) were generated in GraphPad Prism v10.1.1; the heatmap is a schematic representation and does not include numeric coefficients.

Lifestyle and other covariates. Variables from the structured anamnesis (lifestyle, oral hygiene, physical activity, supplementation, medications) were summarized descriptively. Between-group differences for categorical items used χ^2^/Fisher, and for continuous/ordinal items, Welch *t*-tests/ANOVA or nonparametric tests, as appropriate. Given the pilot, exploratory design, and limited sample size, no multivariable adjustment was performed; residual confounding is acknowledged.

Sex-stratified analyses. Pre-specified sex-stratified comparisons were conducted. CIN prevalence (BI ≥ 4.0) was compared separately within women and within men between infertile and fertile cohorts using two-sided Fisher’s exact tests (exact *p*-values). Within-cohort female–male differences were tested with Fisher’s exact tests. Mean BI differences were compared using two-sided Welch’s *t*-tests (with nonparametric sensitivity analyses, Mann–Whitney U/Kruskal–Wallis, where relevant). Effect sizes are reported as RD (percentage points) for prevalence and Hedges’ g for continuous outcomes, each with 95% CIs.

Sensitivity analyses. The primary threshold for cytogenetic fragility was BI ≥ 4.0; robustness was evaluated at BI ≥ 3.5 and BI ≥ 4.5, with interpretations consistent with the primary definition.

## 3. Results

### 3.1. Demographic and Baseline Characteristics

All participants completed the clinical and laboratory evaluations. The mean age of the infertile group (Group A) was 34.7 ± 4.2 years, while the mean age of the fertile control group (Group B) was 33.9 ± 3.8 years, with no statistically significant difference between the two cohorts (*p* = 0.46). Sex distribution was balanced, with 18 women and 12 men in each group. Body mass index (BMI) did not differ significantly between groups (mean ± SD values are reported in Supplementary Table S1). Smoking status and oral hygiene practices also did not differ significantly, indicating comparable baseline risk profiles.

Baseline lifestyle and medication profile. Diet pattern (omnivorous/vegetarian/vegan), oral hygiene routines (toothbrushing frequency, interdental cleaning, mouthrinse use, time since last prophylaxis), physical activity category (sedentary/moderately active/active), supplementation (vitamin D/folate/iron/calcium/multivitamin/probiotics), and 3-month medication history (all Rx/OTC) did not differ significantly between groups (all *p* > 0.05).

### 3.2. Cytogenetic Findings and Chromosomal Instability

Chromosomal Instability. Among the 30 patients in the infertile group, 19 (63.3%) exhibited CIN, defined by a BI ≥ 4.0. The mean BI in this subgroup was 5.2 ± 0.9, whereas the remaining 11 infertile patients without CIN had a significantly lower mean BI of 1.3 ± 0.5. In the fertile control group, all participants displayed normal cytogenetic profiles, with a mean BI of 0.4 ± 0.2 and no metaphases exceeding the pathological threshold.

Sex-stratified analyses. Marked increases in chromosomal instability (CIN) were observed in infertile participants relative to fertile controls in both women and men (see Supplementary Table S2). Women: CIN prevalence (BI ≥ 4.0) was 66.7% in Group A vs. 0.0% in Group B (Fisher’s exact, *p* < 0.0001); mean BI was 3.90 ± 2.06 vs. 0.41 ± 0.20 (Welch’s t, *p* < 0.0001). Men: CIN prevalence was 58.3% vs. 0.0% (Fisher’s exact, *p* < 0.0001); mean BI was 3.58 ± 2.16 vs. 0.39 ± 0.20 (Welch’s t, *p* < 0.0001).

In MMC-stimulated cultures, the observed chromosomal aberrations included chromatid breaks, chromosome gaps, triradial configurations, dicentric chromosomes, and acentric fragments. No numerical abnormalities, such as aneuploidy, were identified in any participant by standard G-banding.

**Figure 1:**
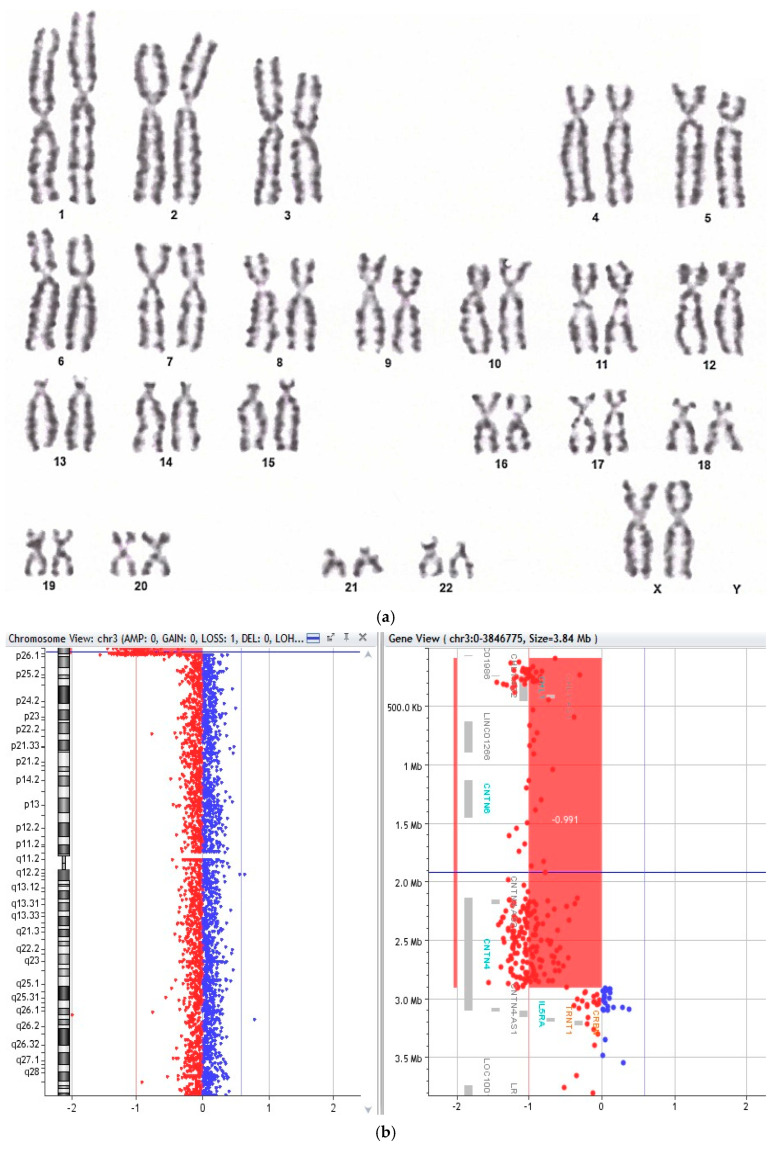
(**a**) Conventional G-banded karyotype (peripheral blood lymphocytes). Karyotype 46,XX,1qh+: benign pericentric heterochromatin variant on chromosome 1q (q12); no other visible numerical or structural abnormalities at 775-band resolution. (**b**) Detail of genetic alterations detected. Array-CGH finding: terminal 3p deletion. Heterozygous loss on 3p26.3–p26.2 (GRCh37: chr3:93,949–2,908,569; ~2.82 Mb), arr[GRCh37] 3p26.3p26.2(93949_2908569)x1. Classified likely pathogenic.

In cases with particularly high BI values or complex aberrations, supplementary array-CGH testing revealed submicroscopic deletions or duplications in 3 out of 19 CIN-positive patients, predominantly involving autosomal loci with potential implications in DNA repair and cell cycle regulation (Figure 1). Y-chromosome microdeletion screening was negative in all male participants.

Within-cohort comparisons showed no statistically significant female–male differences. Infertile (Group A): women vs. men—CIN of 66.7% vs. 58.3% (Fisher’s exact, *p* = 0.712); BI of 3.90 ± 2.06 vs. 3.58 ± 2.16 (Welch’s t, *p* = 0.685). Fertile controls (Group B): women vs. men—CIN of 0.0% vs. 0.0% (Fisher’s exact, *p* = 1.000); BI of 0.41 ± 0.20 vs. 0.39 ± 0.20 (Welch’s t, *p* = 0.791).

### 3.3. Periodontal Status and Disease Severity

Periodontal evaluation demonstrated a significantly higher prevalence and severity of periodontitis among infertile participants with CIN. Particularly, 17 of 19 patients (89.5%) in this subgroup presented with moderate to severe periodontitis, most frequently classified as Stage III according to the 2018 AAP/EFP classification. The mean CAL in this group was 3.2 ± 0.7 mm, and the mean PD was 4.1 ± 0.5 mm. Although the mean PD was 4.1 mm, localized deep pockets and radiographic evidence of bone loss extending into the middle third of the root supported the classification of Stage III periodontitis by the 2018 AAP/EFP criteria.

In contrast, among the 11 infertile patients without CIN, only 6 (54.5%) were diagnosed with periodontitis, primarily of Stage I or II, with a mean CAL of 2.4 ± 0.6 mm and PD of 3.5 ± 0.4 mm. In the control group, periodontitis was identified in 8 individuals (26.7%), all of whom had mild forms (Stage I). The average CAL and PD in this group were 1.7 ± 0.4 mm and 2.2 ± 0.3 mm, respectively.

The distribution of periodontal severity across the groups was statistically significant (χ^2^ = 17.85, *p* < 0.001), suggesting a strong association between CIN and more advanced periodontal disease.

### 3.4. Comparative Table of Clinical and Genetic Parameters

To synthesize the clinical and cytogenetic profiles of the study population, a comparative analysis was performed across the three study groups: infertile individuals with CIN (*n* = 19), infertile individuals without CIN (*n* = 11), and fertile controls (*n* = 30).

As summarized in Table 2A, infertile participants with CIN exhibited a markedly greater periodontal burden than both infertile participants without CIN and fertile controls. The mean BI in this subgroup was 5.2 ± 0.9, indicating chromosomal fragility. This contrasts with the significantly lower BI values in the CIN-negative infertile group (1.3 ± 0.5) and fertile controls (0.4 ± 0.2). Similarly, periodontal indicators revealed a gradient of disease severity corresponding to the presence of CIN: mean CAL was highest in the CIN-positive group (3.2 ± 0.7 mm), followed by the CIN-negative group (2.4 ± 0.6 mm) and controls (1.7 ± 0.4 mm). A comparable trend was observed in PD, PI, and BOP, suggesting a cumulative inflammatory burden in patients with both infertility and genomic instability. To quantify these gradients, Table 2B reports between-group mean differences with 95% confidence intervals (Welch) and standardized effect sizes (Hedges g), together with risk differences (RD) for periodontitis prevalence.

Taken together, these findings strengthen the hypothesis that chronic periodontitis contributes locally and systemically to CIN. They further suggest that comprehensive periodontal evaluation could provide clinically relevant insights in the diagnostic algorithm for patients with idiopathic infertility.

### 3.5. Graphical Representations

**Figure 2:**
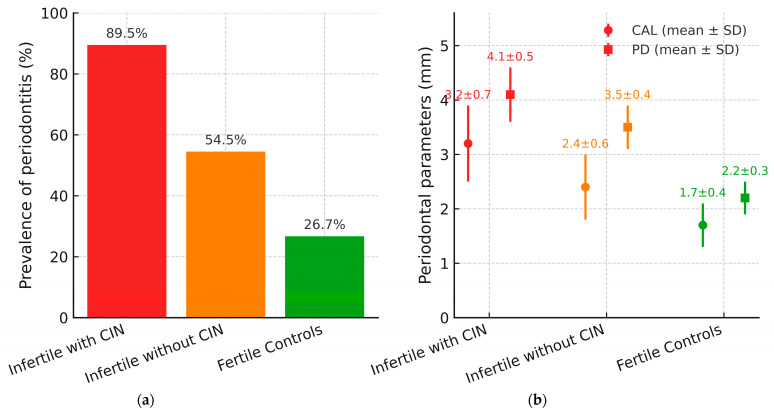
Periodontitis prevalence and periodontal parameters by cohort. (**a**) Prevalence of periodontitis across study groups; bars indicate infertile with CIN (red), infertile without CIN (orange), and fertile controls (green); percentages are shown above the bars. (**b**) Mean (±SD) clinical attachment loss (CAL) and probing depth (PD) by group; colors match panel: circles denote CAL and squares denote PD. Abbreviations: CIN, chromosomal instability; CAL, clinical attachment loss; PD, probing depth; SD, standard deviation.

**Figure 3:**
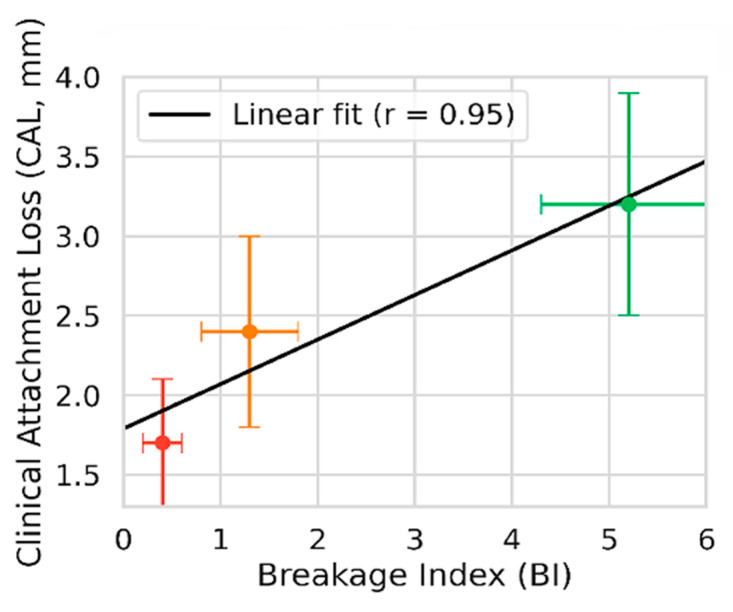
Association between breakage index (BI) and clinical attachment loss (CAL). The scatter depicts the relationship between BI and CAL; colored markers indicate group means (±SD) for infertile with CIN (red), infertile without CIN (orange), and fertile controls (green). Lines, where shown, are visual aids only. Group-wise contrasts are summarized in Table 2. Abbreviations: BI, breakage index; CAL, clinical attachment loss; SD, standard deviation; CIN, chromosomal instability.

**Figure 4:**
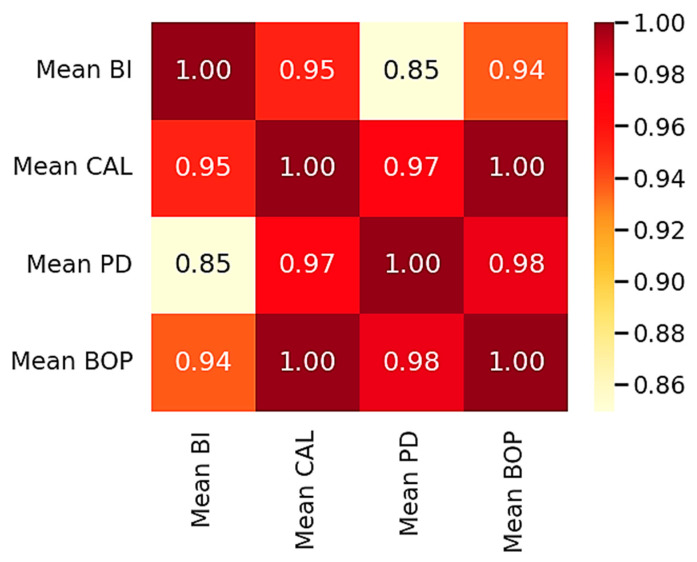
Group-level association matrix among cytogenetic and periodontal parameters. Axes: rows and columns list the four variables—Mean BI, Mean CAL, Mean PD, and Mean BOP. Each cell displays a normalized similarity index (0–1) computed from group-level summaries across the three cohorts (infertile with CIN, infertile without CIN, fertile controls); diagonal cells equal 1.00 by construction. Values serve as a descriptive visual aid and do not represent individual-level correlation coefficients. Darker shading indicates relatively stronger positive association; the color bar indicates the index scale. Abbreviations: BI, breakage index; CAL, clinical attachment loss; PD, probing depth; BOP, bleeding on probing.

To enhance data interpretation and explore inter-variable relationships, graphical analyses are presented in Figure 2, Figure 3 and Figure 4.

As illustrated in Figure 2, the prevalence of periodontitis showed a clear gradient across study groups, with the highest prevalence observed among infertile patients with CIN (89.5%), followed by infertile patients without CIN (54.5%) and fertile controls (26.7%). This pattern supports the association between chromosomal fragility and the severity of periodontal disease.

Figure 3 illustrates the association between the BI and CAL. Patterns are summarized visually, and group-wise contrasts are provided in Table 2.

Collectively, these visual data reinforce the hypothesis that periodontitis may act as a systemic contributor to CIN in individuals diagnosed with idiopathic infertility.

Figure 4 highlights the interdependence between BI and key periodontal indicators. The panel provides a schematic matrix of pairwise associations among BI, CAL, PD, and BOP, conveying the direction and relative strength of the observed gradients across groups.

## 4. Discussion

This study provides new evidence supporting a possible association between CIN and periodontitis in patients with idiopathic infertility. By combining cytogenetic analysis with periodontal assessment, we identified a distinct subgroup of infertile individuals exhibiting both elevated chromosomal fragility and periodontal disease. These findings suggest that the pathophysiological mechanisms of infertility may extend beyond the reproductive system, involving chronic systemic inflammation and genomic instability as contributing factors.

The high frequency of chromosomal aberrations (63.3%) observed in the infertile cohort—particularly among those diagnosed with moderate to severe periodontitis—raises the possibility that chronic oral inflammation may contribute to the onset or aggravation of CIN. Our findings are in line with emerging data demonstrating that persistent inflammatory stimuli, such as those encountered in periodontitis, can compromise genomic integrity through several biological pathways [27,34,35].

One of the most convincing mechanisms linking periodontitis and CIN involves oxidative stress [36,37]. Periodontal tissues chronically exposed to bacterial biofilms produce large quantities of ROS, including superoxide anions, hydrogen peroxide, and hydroxyl radicals [38,39]. These reactive species are capable of inducing single- and double-strand DNA breaks, base modifications, and cross-linking, ultimately leading to chromosomal aberrations if not adequately repaired [40,41]. In addition to direct DNA damage, ROS may also trigger mitochondrial dysfunction and impair germinal cell energy metabolism, further compromising gametogenesis [42,43]. Oxidative DNA damage is a hallmark of both chronic inflammatory conditions and impaired gametogenesis [44,45].

Additionally, the systemic spillover of pro-inflammatory cytokines—such as interleukin-1β, interleukin-6, tumor necrosis factor-alpha (TNF-α), and C-reactive protein—has been shown to impair the function of DNA repair enzymes and disrupt the mitotic spindle apparatus [46]. This chronic low-grade inflammation, often referred to as “inflammaging,” may create a persistent genotoxic microenvironment that affects not only somatic cells but also germline integrity [47,48]. These cytokines are elevated in both chronic periodontitis and subfertile individuals, and may collectively enhance the likelihood of chromosomal missegregation, breakage, or aneuploidy during germ cell division [49,50,51].

Furthermore, it is noteworthy that telomere attrition, a marker of cellular aging and genomic instability, is accelerated in patients with periodontitis [52]. Telomeres serve as protective caps at the ends of chromosomes, and their progressive shortening in inflammatory environments renders chromosomes more vulnerable to structural damage and fusion events [53,54]. Emerging evidence also links telomere dysfunction with poor spermatogenesis and decreased oocyte quality, suggesting that periodontal inflammation may indirectly influence reproductive capacity via telomere-mediated mechanisms [55]. Thus, periodontitis could serve as both a marker and a driver of CIN in susceptible individuals [56].

Quantitatively, we observed robust associations between cytogenetic instability and periodontal status. The BI correlated strongly and positively with CAL, PD, and BOP. These correlations, visualized through scatter plots and heatmaps, support the biological plausibility of a link between the periodontal and reproductive systems mediated through genomic instability. Such findings support a “systems biology” perspective, wherein oral infections contribute to reproductive dysfunction through shared pathways of oxidative stress, DNA damage, and immune dysregulation [57,58,59].

Notwithstanding these associations, contextual determinants merit consideration. Socioeconomic status (SES), access to healthcare, and lifestyle or environmental stressors may influence both periodontal status and genomic stability. Although baseline lifestyle indicators did not differ significantly between cohorts, the present study did not capture a formal SES index or detailed environmental exposures; therefore, residual confounding cannot be excluded. Accordingly, periodontitis should be interpreted as a potential marker of broader, chronic exposures rather than a sole proximal cause of CIN in this cross-sectional design. This interpretation aligns with a multifactorial framework in which oral inflammation overlaps with social and environmental determinants that collectively shape systemic inflammatory tone and genome integrity.

The findings of this study align with prior investigations reporting increased sperm DNA fragmentation in men with chronic periodontitis, and with studies indicating that women with poor oral health are at higher risk for adverse pregnancy outcomes [60,61]. However, to our knowledge, this is the first study to directly correlate chromosomal breakage with periodontal status in the context of idiopathic infertility. This integrative approach supports the systemic implications of oral disease and introduces a new perspective on its potential role in idiopathic infertility.

From a clinical standpoint, these results support a larger, interdisciplinary approach to infertility evaluation, that incorporates periodontal screening and management of periodontal disease. Addressing modifiable risk factors such as oral inflammation may improve systemic health and potentially restore reproductive competence in some individuals.

In summary, our findings suggest that periodontitis may contribute to the pathogenesis of idiopathic infertility by facilitating CIN. This new connection between oral health and reproductive health underlines the need for integrated care strategies and opens up new avenues for research into the systemic effects of chronic inflammation.

## 5. Limitations

This exploratory, single-center, cross-sectional study has a modest sample size, which limits precision, power (especially for sex-stratified analyses), and generalizability. Although baseline lifestyle indicators did not differ materially, we did not collect a formal SES index or detailed environmental exposures; residual confounding cannot be excluded, and no multivariable adjustment was performed. Mechanistic interpretation is limited by the absence of systemic biomarkers (e.g., cytokines, oxidative stress markers, telomere metrics) and by the use of MMC-induced lymphocyte fragility as the sole genomic assay. Analyses followed a prespecified plan emphasizing group-wise contrasts with 95% CIs, with sensitivity checks for BI thresholds (≥3.5 and ≥4.5); results should be considered hypothesis-generating.

## 6. Suggestions for Future Directions

Future research should focus on:

- Causality and trials: Prospective longitudinal cohorts and preregistered randomized trials testing whether standardized periodontal therapy lowers BI and improves reproductive outcomes (time-to-pregnancy, ART success, live birth).
- Bias control: Collection of validated SES and environmental exposure measures, coupled with causal-inference methods (propensity scores, DAGs, marginal structural models).
- Mechanisms and standardization: Evaluation of dose–response and mediation via inflammatory/oxidative biomarkers and telomere metrics, and multicenter studies with harmonized periodontal protocols, examiner calibration, centralized cytogenetics, and transparent plans (core outcomes, a priori sample size, data/code sharing).

## 7. Conclusions

This study documents a cross-sectional association between chromosomal instability (CIN) and periodontitis in idiopathic infertility. Using integrated cytogenetic testing and comprehensive periodontal examination, infertile participants with CIN exhibited a higher prevalence and greater severity of periodontitis than infertile participants without CIN and fertile controls; gradients in BI aligned consistently with CAL, PD, and BOP.

Because the design is cross-sectional and formal socioeconomic indices and detailed environmental exposures were not collected, causal inference is not warranted, and residual confounding cannot be excluded. Periodontitis should therefore be considered a potential marker—and plausible contributor—within a multifactorial framework linking systemic inflammation and genome integrity.

Clinically, incorporating periodontal screening and management into multidisciplinary infertility care is feasible, non-invasive, and potentially cost-effective, with the prospect of improving overall health. At the same time, hypotheses regarding reproductive outcomes are tested. Prospective cohort and preregistered interventional studies—ideally multicenter, sex-stratified, and biomarker-enriched (e.g., telomere length, oxidative DNA damage markers, cytokines) and adjusted for socioeconomic and environmental determinants—are warranted to confirm these links and clarify mechanisms.

## Figures and Tables

**Table 1:** Inclusion and exclusion criteria.

Parameter	Inclusion Criteria	Exclusion Criteria
Age	20–40 years	<20 or >40 years
Cases (Group A)	Idiopathic infertility: ≥12 months of regular unprotected intercourse with no identifiable anatomical, hormonal, infectious, or endocrinological cause	Identified cause of infertility or duration <12 months
Controls (Group B)	Fertile: natural conception within the last 3 years; no history of infertility	History of infertility/subfertility or conception via assisted reproduction among controls
Smoking	Non-smoker or light smoker (<10 cigarettes/day)	Heavy smoking (≥10 cigarettes/day)
Systemic status	No chronic systemic inflammatory disease (e.g., diabetes mellitus, autoimmune disease, inflammatory bowel disease)	Presence of a chronic systemic inflammatory disease
Pregnancy/ Lactation	Not pregnant and not lactating	Pregnancy or lactation
Chromosomal disorders	No known chromosomal disorder	Known chromosomal disorder
Immunosuppressive therapy	No immunosuppressive therapy currently or within the past 3 months	Immunosuppressive therapy currently or within the past 3 months
Malignancy	No active malignancy/no ongoing oncologic treatment (remote history in complete remission allowed)	Active malignancy
Medications affecting periodontal tissues, bone, or immunity (≤3 months)	None of the following in the past 3 months	Any of the following in the past 3 months: systemic corticosteroids; calcineurin inhibitors (cyclosporine, tacrolimus); antineoplastic agents; antiresorptives (bisphosphonates, denosumab); calcium-channel blockers associated with gingival overgrowth (e.g., nifedipine); phenytoin; oral retinoids
Systemic antibiotics/NSAIDs (≤30 days)	No systemic antibiotics or NSAIDs within the previous 30 days	Systemic antibiotics or NSAIDs within the previous 30 days (temporary exclusion; rescreen after a 30-day washout)
Periodontal therapy (≤6 months)	No periodontal therapy within the previous 6 months	Periodontal therapy within the previous 6 months

**Table 2:** Comparative overview of clinical and cytogenetic characteristics among infertile patients with and without CIN and fertile controls.

(A) Group Summaries (*n*, Mean ± SD)				
Parameter		Infertile with CIN (*n* = 19)	Infertile Without CIN (*n* = 11)	Fertile Controls (*n* = 30)
Mean Breakage Index (BI)		5.2 ± 0.9	1.3 ± 0.5	0.4 ± 0.2
Periodontitis Prevalence		89.5% (17/19)	54.5% (6/11)	26.7% (8/30)
Mean CAL (mm)		3.2 ± 0.7	2.4 ± 0.6	1.7 ± 0.4
Mean Probing Depth (mm)		4.1 ± 0.5	3.5 ± 0.4	2.2 ± 0.3
Mean Plaque Index		2.3 ± 0.4	1.9 ± 0.5	1.4 ± 0.3
Mean BOP (% sites)		35.2 ± 8.6	26.4 ± 7.1	17.3 ± 6.8
**(B) Between-group contrasts (mean difference with 95% CI; Hedges g)**				
**Metric**	**Comparison**	**Mean** **Difference**	**95% CI**	**Hedges g**
CAL (mm)	CIN vs. NoCIN	0.80	0.30 to 1.30	1.17
	CIN vs. Controls	1.50	1.14 to 1.86	2.76
	NoCIN vs. Controls	0.70	0.27 to 1.13	1.49
PD (mm)	CIN vs. NoCIN	0.60	0.30 to 0.90	1.35
	CIN vs. Controls	1.90	1.64 to 2.16	4.94
	NoCIN vs. Controls	1.30	1.07 to 1.53	3.38
Plaque Index	CIN vs. NoCIN	0.40	0.03 to 0.77	—
	CIN vs. Controls	0.90	0.68 to 1.12	—
	NoCIN vs. Controls	0.50	0.15 to 0.85	—
BOP (% sites)	CIN vs. NoCIN	+8.8 pp	+2.73 to +14.87	—
	CIN vs. Controls	+17.9 pp	+13.14 to +22.66	—
	NoCIN vs. Controls	+9.1 pp	+3.83 to +14.37	—
Periodontitis prevalence (RD)	CIN vs. NoCIN	+0.35	−0.10 to +0.69	—
	CIN vs. Controls	+0.63	+0.24 to +0.83	—
	NoCIN vs. Controls	+0.28	−0.16 to +0.65	—

Notes (A): Classification of periodontitis severity was based on the 2018 AAP/EFP criteria. Only Stage I–III were identified; no cases met Stage IV; Notes (B): pp = percentage points; CI = 95% confidence interval using Welch (continuous outcomes) and Newcombe (risk differences). Hedges g is reported for CAL and PD.

## Data Availability

The data presented in this study are available on request from the corresponding author.

## Supplementary Materials

The following supporting information can be downloaded at: https://www.mdpi.com/article/10.3390/biology14091256/s1, Table S1: Categorical Variables; Table S2: Sex-stratified chromosomal instability (CIN) by cohort.

## Abbreviations

The following abbreviations are used in this manuscript:

AAP	American Academy of Periodontology
aCGH	Array-based Comparative Genomic Hybridization
BI	Breakage Index
BOP	Bleeding on Probing
BMI	Body Mass Index
CAL	Clinical Attachment Loss
CIN	Chromosomal Instability
CI	Confidence Interval
DNA	Deoxyribonucleic Acid
EFP	European Federation of Periodontology
ICC	Intraclass Correlation Coefficient
IL	Interleukin
IRB	Institutional Review Board
IQR	Interquartile Range
MMC	Mitomycin C
NSAID	Nonsteroidal Anti-Inflammatory Drug
OTC	Over-the-Counter
PCR	Polymerase Chain Reaction
PD	Probing Depth
PI	Plaque Index
RD	Risk Difference
ROS	Reactive Oxygen Species
SD	Standard Deviation
SES	Socioeconomic Status
SPSS	Statistical Package for the Social Sciences
TNF-α	Tumor Necrosis Factor-alpha
UNC-15	University of North Carolina-15 periodontal probe
