# Supplementary material for: Chromosomal Instability and Periodontal Disease in Idiopathic Infertility: Evidence of a Possible Association
Source: Biology (Basel). 2025 Sep 12;14(9):1256. doi: 10.3390/biology14091256 (PMC12467813; doi:10.3390/biology14091256)
Supplement: Supplementary file 1 [file biology-14-01256-s001.zip › biology-3844240-supplementary.pdf]

**Supplementary Table S1.** Categorical Variables

| Variable | Group A (Infertile) | Group B (Fertile controls) | p-value |
|---|---|---|---|
| Female, n (%) | 18 (60.0%) | 18 (60.0%) | — |
| Male, n (%) | 12 (40.0%) | 12 (40.0%) | — |
| Current smokers, n (%) | 5 (16.7%) | 5 (16.7%) | 1.000 |
| Toothbrushing ≥2/day, n (%) | 23 (76.7%) | 24 (80.0%) | 1.000 |
| Interdental cleaning (any), n (%) | 12 (40.0%) | 13 (43.3%) | 1.000 |
| Mouthrinse use ≥1/week, n (%) | 14 (46.7%) | 15 (50.0%) | 1.000 |
| Last prophylaxis ≤12 months, n (%) | 20 (66.7%) | 22 (73.3%) | 0.778 |
| Physical activity: sedentary / moderately active / active, n (%) | 9 / 14 / 7 | 8 / 15 / 7 | 0.954 |
| Any supplementation, n (%) | 17 (56.7%) | 18 (60.0%) | 1.000 |
| Any medication in past 3 months, n (%) | 11 (36.7%) | 10 (33.3%) | 1.000 |

Notes: Values are n (%). Between-group differences were evaluated with $\chi^2$ or Fisher's exact tests, as appropriate; exact two-sided p-values are shown. Abbreviation: —, not applicable.

**Supplementary Table S2.** Sex-stratified chromosomal instability (CIN) by cohort

Prevalence of CIN (BI ≥ 4.0) and mean BI ± SD are presented separately for women and men in each cohort. Prevalence is compared using two-sided Fisher's exact tests; mean BI is compared using two-sided Welch's t-tests. Exact two-sided p-values are reported.

| **Cohort** | **Women n** | **Women: CIN ≥ 4.0 n (%)** | **Women: BI (mean ± SD)** | **Men n** | **Men: CIN ≥ 4.0 n (%)** | **Men: BI (mean ± SD)** | **p (prevalence)*** | **p (BI)*** |
|---|---|---|---|---|---|---|---|---|
| Infertile (Group A) | 18 | 12 (66.7%) | 3.90 ± 2.06 | 12 | 7 (58.3%) | 3.58 ± 2.16 | 0.712 | 0.685 |
| Fertile controls (Group B) | 18 | 0 (0.0%) | 0.41 ± 0.20 | 12 | 0 (0.0%) | 0.39 ± 0.20 | 1.000 | 0.791 |

*p-values refer to female–male comparisons within the same cohort.
